# Active Time-Restricted Feeding Improved Sleep-Wake Cycle in *db/db* Mice

**DOI:** 10.3389/fnins.2019.00969

**Published:** 2019-09-20

**Authors:** Tianfei Hou, Chanung Wang, Shreyas Joshi, Bruce F. O’Hara, Ming C. Gong, Zhenheng Guo

**Affiliations:** ^1^Department of Physiology, University of Kentucky, Lexington, KY, United States; ^2^Department of Biology, University of Kentucky, Lexington, KY, United States; ^3^Department of Pharmacology and Nutritional Sciences, University of Kentucky, Lexington, KY, United States; ^4^Research and Development, Lexington Veterans Affairs Medical Center, Lexington, KY, United States

**Keywords:** active time-restricted feeding, sleep, circadian rhythm, diabetes, *db/db* mice

## Abstract

People with diabetes are more likely to experience sleep disturbance than those without. Sleep disturbance can cause daytime sleepiness in diabetic patients, which may impair their daytime performance or even lead to workplace injuries. Therefore, restoring the normal sleep-wake cycle is critical for diabetic patients who experience daytime sleepiness. Previous data on a diabetic mouse model, the *db/db* mice, have demonstrated that the total sleep time and sleep fragmentation are increased and the daily rhythm of the sleep-wake cycle is attenuated. Accumulating evidence has shown that active time-restricted feeding (ATRF), in which the timing of food availability is restricted to the active-phase, is beneficial to metabolic health. However, it is unknown whether ATRF restores the normal sleep-wake cycle in diabetes. To test that, we used a non-invasive piezoelectric system to monitor the sleep-wake profile in the *db/db* mice with *ad libitum* feeding (ALF) as a baseline and then followed with ATRF. The results showed that at baseline, *db/db* mice exhibited abnormal sleep-wake patterns: the sleep time percent during the light-phase was decreased, while during the dark-phase it was increased with unusual cycling compared to control mice. In addition, the sleep bout length during both the light-phase and the full 24-h period was shortened in *db/db* mice. Analysis of the sleep-wake circadian rhythm showed that ATRF effectively restored the circadian but suppressed the ultradian oscillations of the sleep-wake cycle in the *db/db* mice. In conclusion, ATRF may serve as a novel strategy for treating diabetes-induced irregularity of the sleep-wake cycle.

## Introduction

The prevalence of diabetes has been increasing over the past few decades. Over 400 million adults were estimated to have diabetes worldwide in 2017 (IDF Diabetes Atlas, 2017). It is well-documented that sleep disorders contribute to the development of diabetes (Cappuccio et al., 2010). Recent evidence has shown that the relationship between diabetes and sleep disorders is bidirectional (Tasali et al., 2008). Epidemiological studies have shown that insomnia, daytime sleepiness, prolonged sleep latency, and poor sleep maintenance is more frequent in diabetic patients than non-diabetic subjects (Lamond et al., 2000; Skomro et al., 2001; Nakanishi-Minami et al., 2012; Plantinga et al., 2012; Medeiros et al., 2013; Lecube et al., 2016). Daytime sleepiness such as that caused by sleep disturbance in diabetic patients may compromise daytime performance or even lead to workplace accidents (Horne and Reyner, 1995; Melamed and Oksenberg, 2002). Therefore, restoring the normal sleep-wake cycle and reducing daytime sleepiness is important for the life quality, daytime performance, and work safety of diabetic patients who experience daytime sleepiness.

The sleep-wake cycle is regulated by multiple factors, among which the circadian rhythm is one of the most important factors (Eban-Rothschild et al., 2018). A large body of evidence demonstrates that the timing of food intake plays a prominent role in entraining circadian clocks in peripheral tissues. When nocturnal animals are fed only during the inactive light-phase, the phase of clock gene oscillations in various tissues are reversed/shifted (Damiola et al., 2000; Stokkan et al., 2001). In contrast, when the feeding time is restricted to the active dark-phase in the nocturnal rodents, the disrupted clock gene oscillations are restored with high-fat diet- or genetic mutant-induced obese/diabetic rodents (Kudo et al., 2004; Hatori et al., 2012). In addition, active-phase time-restricted feeding (ATRF) also improves metabolic dysregulation in diabetes (Mistlberger et al., 1998; Arble et al., 2009; Hatori et al., 2012; Sherman et al., 2012; Tsai et al., 2013; Adamovich et al., 2014; Chaix et al., 2014; Yasumoto et al., 2016). However, it is unknown whether ATRF is able to improve the sleep-wake cycle in diabetic humans or other animal models of diabetes.

The leptin receptor mutant *db/db* mouse is a commonly used type 2 diabetic mouse model, which manifests syndromes that resemble maturity-onset diabetes in humans (Ktorza et al., 1997). The *db/db* mice exhibit a variety of alterations in sleep-wake architecture, including increased total sleep time in 24-h, mostly due to increased sleep time during the active dark-phase. In addition, sleep fragmentation is elevated (Laposky et al., 2008). To test whether ATRF improves sleep-wake cycles in diabetes, we monitored the sleep-wake states of the *db/db* mice with *ad libitum* feeding (ALF) and ATRF by using a non-invasive PiezoSleep system. Our results demonstrated that ATRF was able to improve the sleep-wake patterns in *db/db* mice to a state that is comparable to control mice.

## Method

### Animals

Male *db/db* and control (*db/*+) *C57BL/KsJ* mice (*n* = 8 in each strain) were purchased from the Jackson lab (Stock No. 000642). After arrival and throughout the experiment, the mice were housed in a 12L: 12D light: dark cycle with lights on from 9 pm to 9 am. Normal chow diet and water were provided *ad libitum*.

### Experimental Procedure

The sleep-wake states were determined in 15-week-old control and *db/db* mice using a piezoelectric system. Prior to data collection, the mice were acclimated in the system for 4 weeks. The baseline sleep-wake states were recorded for 5 consecutive days in control and *db/db* mice with ALF. Then the mice were subjected to an ATRF regimen for a total of 18 days, during which sleep was monitored day 1–5 and day 15–17. The ATRF was administered by quietly manually adding food at ZT13 (10 am) and removing food at ZT21 (6 pm) by the same investigator. The disturbance caused by the food addition or removal was brief (<1 min for each mouse). Sham interaction between the investigator with the mice under ALF regimen was not performed because each mouse was fed ALF initially and then switched at the same time to ATRF. The water was given *ad libitum*. During the over 3 weeks of sleep recording, some data with artifacts were excluded: 2 control and 1 *db/db* mice at baseline and 2 *db/db* mice for day 1–5. The different numbers of the exclusion during different recording periods were due to the re-setup of the recording system when cleaning the cages. All animal procedures were approved by the Institutional Animal Care and Use Committee.

### Body Weight and Blood Glucose Measurement

The body weight and non-fasting blood glucose were measured before starting the ATRF and after 18 days of ATRF, both at ZT20. To measure non-fasting blood glucose, the mouse tail was sniped to obtain a drop of blood. Then a disposable test strip (StatStrip Xepress^TM^ glucometer, NOVA Biomedical; Waltham, MA, United States) was used to measure the glucose level in the blood drop.

### The Piezoelectric System

The piezoelectric system is a non-invasive, highly sensitive motion detector system, as described previously (Flores et al., 2007; Mang et al., 2014; Yaghouby et al., 2016) and at www.sigsoln.com (Signal Solutions LLC, Lexington, KY, United States). In brief, each cage unit housed a single mouse inside 18-cm × 18-cm walled compartments with attached food and water structures. The cages had open bottoms resting on polyvinylidene difluoride (PVDF) sensors serving as the cage floor. The PVDF sensors were coupled to an input differential amplifier, and pressure signals were generated and classified as motions consistent with either activity related to wake, or inactivity and regular breathing movements associated with sleep. Sleep was characterized primarily by periodic (2–3 Hz) and regular amplitude signals, which is typical of respiration from a sleeping mouse. In contrast, signals characteristic of wake were both the absence of characteristic sleep signals and higher amplitude, and irregular signals were associated with volitional movements, even subtle head movements during quiet wake. The piezoelectric signals in 2-s epochs were classified by a linear discriminant classifier algorithm based on multiple signal variables to assign a binary label of “sleep” or “wake.” Data collected from the cage system were binned over specified time periods (e.g., 1 h) using the average of percent sleep as well as by length of individual bouts of sleep.

### Non-parametric Circadian Rhythm Analysis

The daily rhythm of wake percent was analyzed by a non-parametric circadian rhythm analysis method using the Clocklab analysis software (van Someren et al., 1996). The non-parametric circadian rhythm method measures several parameters, including interdaily stability (IS), intradaily variability (IV), L5 average and L5 start time, M10 average and M10 start time, and relative amplitude (RA). IS quantifies the strength of resemblance from 1 day to the next; IV measures the fragmentation between rest and activity; L5 average and L5 start are the average wake time and the start time of the 5-h period with the least wake time; M10 average and M10 start are the average wake time and the start time of the 10-h period with the most wake time; and RA compares the periods of lowest and highest wake time: RA = (M10 Avg – L5 Avg)/(M10 Avg + L5 Avg).

### Statistical Analysis

The statistical analysis was performed using GraphPad Prism (version 8). Two-way ANOVA with repeated measurement with Geisser-Greenhouse correction and Sidak *post hoc* test was used. A mixed-effects model was performed if there are missing data. The data were expressed as mean ± SEM. A *p* value less than 0.05 was considered statistically significant.

## Results

### The Sleep-Wake Pattern Was Altered in the *db/db* Mice With *ad libitum* Feeding (ALF)

**FIGURE 1:**
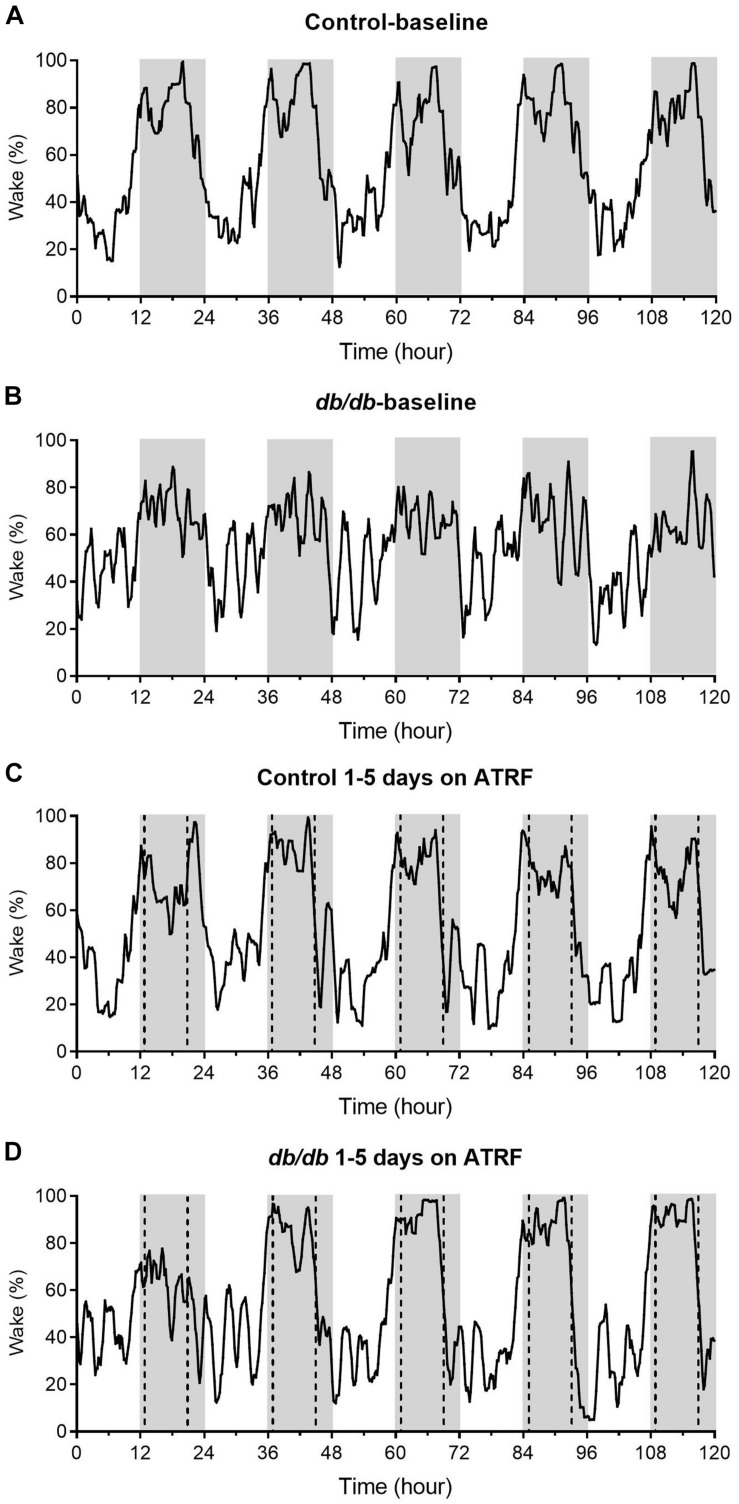
Representative sleep-wake profiles of the control and *db/db* mice with ALF and the 1–5 days of ATRF. The sleep-wake profiles in the control mice with ALF **(A)** or ATRF **(C)** and in the *db/db* mice with ALF **(B)** or ATRF **(D)**. The gray box indicates the dark-phase. The dotted vertical line in **C,D** indicates the time when food was added or withdrawn during the ATRF feeding paradigm.

We monitored the sleep-wake profile in groups of *db/db* and control mice with ALF as baseline and then switched to ATRF to determine the effects of ATRF on the sleep-wake profile of the diabetic *db/db* mice. As shown in the representative recordings (Figure 1) and the mean with 95% confidence interval plots (Supplementary Figure S1), at baseline with ALF, the daily sleep profile in the *db/db* mice (Figure 1B and Supplementary Figure S1B) was significantly different from the control mice (Figure 1A and Supplementary Figure S1A). In contrast to the clear and large difference in the wake percent between the light- and dark-phase, the percent waking time of the *db/db* mice in either the light- or dark-phase fluctuated with a sawtooth-like pattern (Figure 1B) and the difference between the light- and dark-phase seems to be smaller compared to the controls.

**FIGURE 2:**
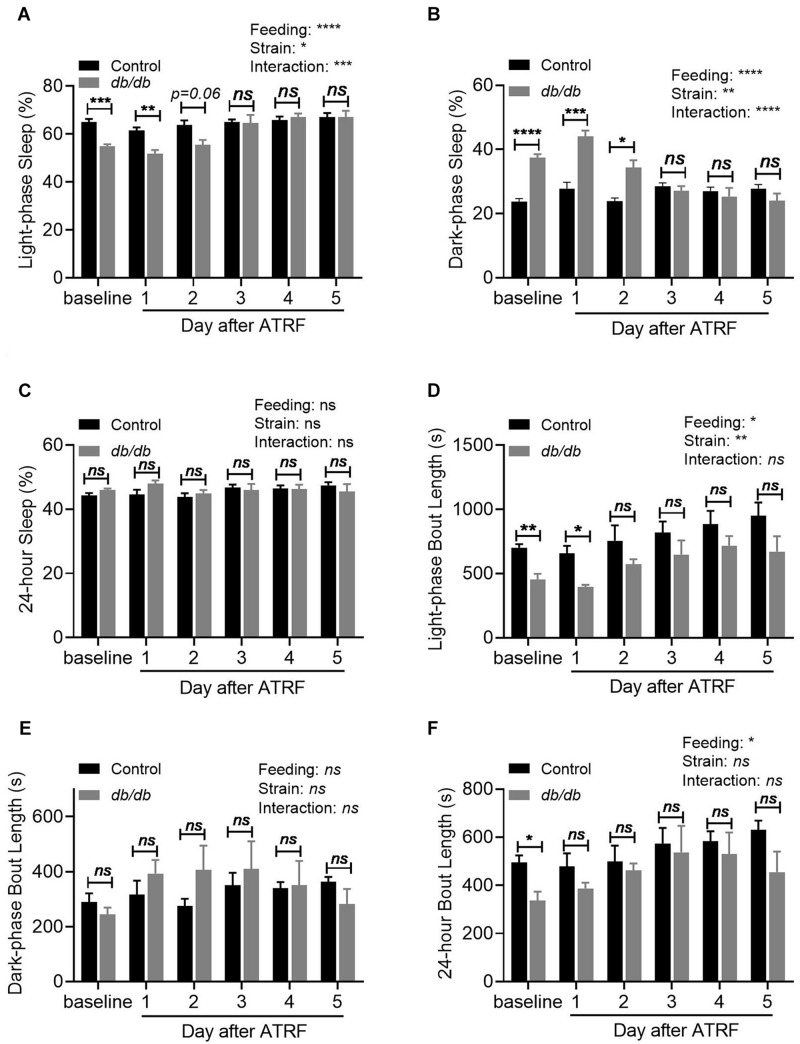
Quantification of the sleep-wake profile of the control and *db/db* mice with ALF and the 1–5 days of ATRF. **(A–C)** Average sleep time percent during the light-phase **(A)**, dark-phase **(B),** and over 24-h **(C)**. **(D–F)** Average sleep bout length during the light-phase **(D)**, dark-phase **(E)**, and over 24-h **(F)**. Control, *n* = 6–8; *db/db*, *n* = 6–7. ^∗^*p* < 0.05; ^∗∗^*p* < 0.01; ^∗∗∗^*p* < 0.001; ^****^p < 0.0001; *ns*, no significant.

Quantification of the sleep time showed that the sleep percent during the light-phase was significantly decreased from 64.9 ± 3.33% in the control to 54.8 ± 2.56% in the *db/db* mice (*p* < 0.01, Figure 2A and Supplementary Table S1). In contrast, the sleep percent during the dark-phase was significantly increased from 23.7 ± 2.39% in the control to 37.5 ± 2.77% in the *db/db* mice (*p* < 0.001, Figure 2B and Supplementary Table S1). With a decrease in the light-phase and an increase in the dark-phase sleep percent, the 24-h sleep percent showed no difference between the control and *db/db* mice (*p* > 0.05, Figure 2C and Supplementary Table S1). For the sleep bout length, there was a significant decrease during the light-phase from 701.5 ± 68.57 s in the control to 455.2 ± 113.5 s in the *db/db* mice (*p* < 0.01, Figure 2D and Supplementary Table S2). There was no change during the dark-phase (*p* > 0.05, Figure 2E and Supplementary Table S2), which resulted in a significant decrease in the 24-h sleep bout length from 495.7 ± 69.43 s in the control to 337.0 ± 97.26 s in the *db/db* mice (*p* < 0.05, Figure 2F and Supplementary Table S2).

### Active Time-Restricted Feeding (ATRF) Improved the Sleep-Wake Pattern in the *db/db* Mice

The sleep-wake profile showed that ATRF did not alter the sleep-wake pattern in the control mice (Figure 1C vs. Figure 1A and Supplementary Figure S1A). However, ATRF gradually improved the pattern in the *db/db* mice to resemble the control mice (Figure 1D vs. Figure 1B and Supplementary Figure S1B). By the third day of ATRF, the sleep-wake pattern in the *db/db* mice was similar to that in the control mice (Figure 1D and Supplementary Figure S1B).

Quantification of the sleep time showed that the *db/db* mice still exhibited lower light-phase sleep percent than the control mice on the first and second days of ATRF but increased to the same level as that of the control mice by the third day of ATRF (Figure 2A and Supplementary Table S1). ATRF significantly increased the light-phase sleep percent in the *db/db* mice (baseline vs. fifth day of ATRF: *p* < 0.001), but did not affect the light-phase sleep percent in the control mice. The dark-phase sleep percent in the *db/db* mice remained higher than in controls on the first two days of ATRF but was equivalent to that of control mice by the third day of ATRF (Figure 2B and Supplementary Table S1). The dark-phase sleep percent was not significantly different in the control mice between baseline vs. the fifth day of ATRF; however, it was significantly decreased in the *db/db* mice (*p* < 0.0001). The 24-h sleep percent was not significantly different between *db/db* and control mice or between baseline vs. ATRF (Figure 2C and Supplementary Table S1). The decrease of the sleep bout length during the light-phase (Figure 2D and Supplementary Table S2) or the 24 h (Figure 2F and Supplementary Table S2) in the *db/db* mice was abolished by the ATRF on the 2nd and 1st day, respectively.

### ATRF Significantly Improved the Daily, but Suppressed the Ultradian Sleep-Wake Oscillations in the *db/db* Mice

**FIGURE 3:**
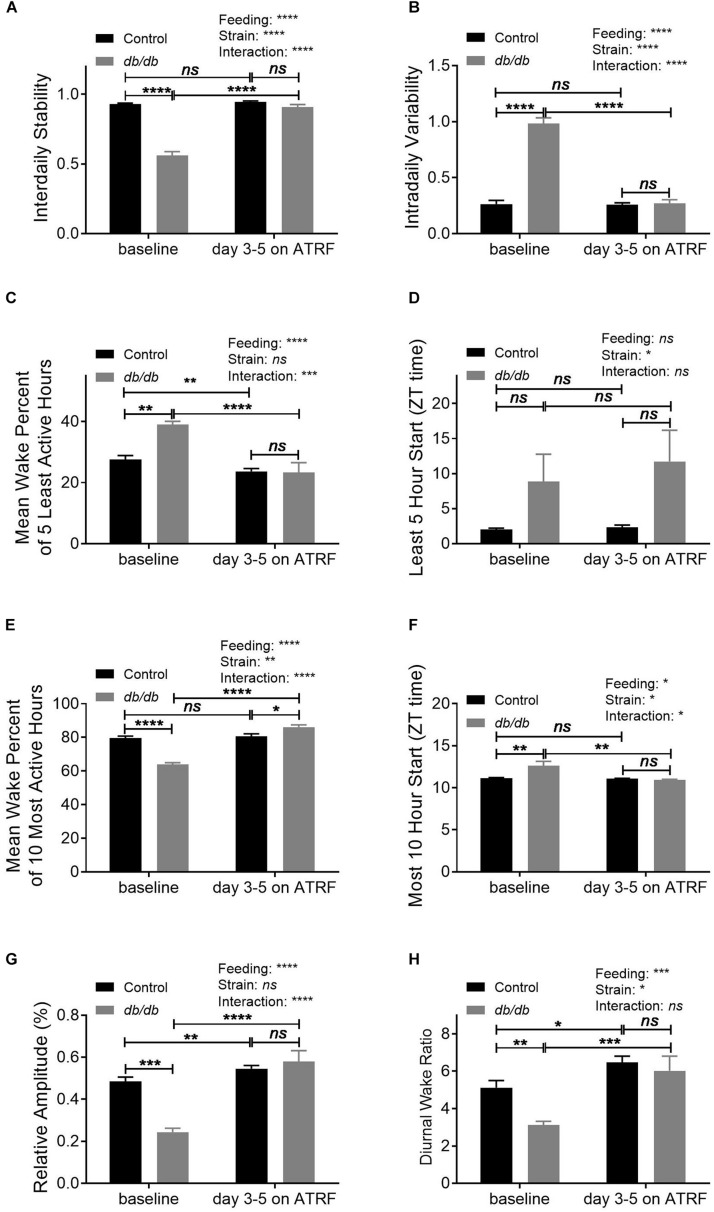
Non-parametric circadian rhythm analysis of the sleep-wake cycles of the control and *db/db* mice with ALF and the 1–5 days of ATRF. The non-parametric circadian rhythm analysis was performed using Clocklab analysis software. **(A)** Interdaily stability, **(B)** intradaily variability, **(C,D)** mean wake percent of the 5 least wake hours **(C)** and start time **(D)**, **(E,F)** mean wake percent of 10 most wake hours **(E)** and start time **(F)**, **(G)** relative amplitude, and **(H)** diurnal wake ratio calculated by the Piezoelectric system. Control, *n* = 6–8; *db/db*, *n* = 6–7. ^∗^*p* < 0.05; ^∗∗^*p* < 0.01; ^∗∗∗^*p* < 0.001; ^****^p < 0.0001; *ns*, no significant.

To further characterize the daily oscillation of the sleep-wake cycle, we analyzed its pattern using a non-parametric method. The results showed that the IS was significantly decreased (Figure 3A and Supplementary Table S3) whereas the IV was significantly increased (Figure 3B and Supplementary Table S3) in the *db/db* mice compared to control mice under baseline with ALF feeding. The ATRF effectively restored both the IS and IV in the *db/db* mice to the control level (Figures 3A,B and Supplementary Tables S3, S4). During the least awake 5-h period in 1 day, the *db/db* mice had a higher mean wake percent at the baseline (Figure 3C and Supplementary Table S3). ATRF effectively suppressed it to the control level (Figure 3C and Supplementary Tables S3, S4) without changing the delayed starting time of the least awake 5-h (Figure 3D and Supplementary Table S4). For the most awake 10-h period in 1 day, the *db/db* mice had a decreased mean wake percent (Figure 3E and Supplementary Table S3). ATRF increased it to a level even higher than in the control mice (Figure 3E and Supplementary Tables S3, S4). The delayed starting time of the most awake 10-h period in 1 day in the *db/db* mice was restored to the controls (Figure 3F and Supplementary Tables S3, S4). The RA of the daily oscillation (Figure 3G and Supplementary Tables S3, S4) and the ratio of dark/light-phase wake time (Figure 3H and Supplementary Tables S3, S4) were suppressed in the *db/db* under ALF and restored to control level with ATRF.

While the near 24-h daily oscillation was the dominant rhythm in both the control and *db/db* mice, the amplitude of the daily oscillation was compromised in the *db/db* mice (Supplementary Figure S2A vs. Supplementary Figures S2B,E and Supplementary Table S3) and restored by ATRF (Supplementary Figure S2C vs. Supplementary Figures S2D,E and Supplementary Tables S3, S4). In contrast, there was an increase in the ultradian oscillations in the *db/db* mice at baseline, especially oscillations with period length shorter than 6 h (Supplementary Figure S2B vs. Supplementary Figures S2A,F and Supplementary Table S3). Impressively, ATRF suppressed the ultradian oscillations of *db/db* mice to the control level (Supplementary Figure S2D vs. Supplementary Figures S2C,F and Supplementary Tables S3, S4). Taken together, ATRF was effective in restoring the daily and suppressing the ultradian oscillations in the *db/db* mice to the control level.

### The Improvement of the Sleep-Wake Cycle in the *db/db* Mice Induced by ATRF Persisted Through 17 Days and Was Associated With Body Weight Loss

**FIGURE 4:**
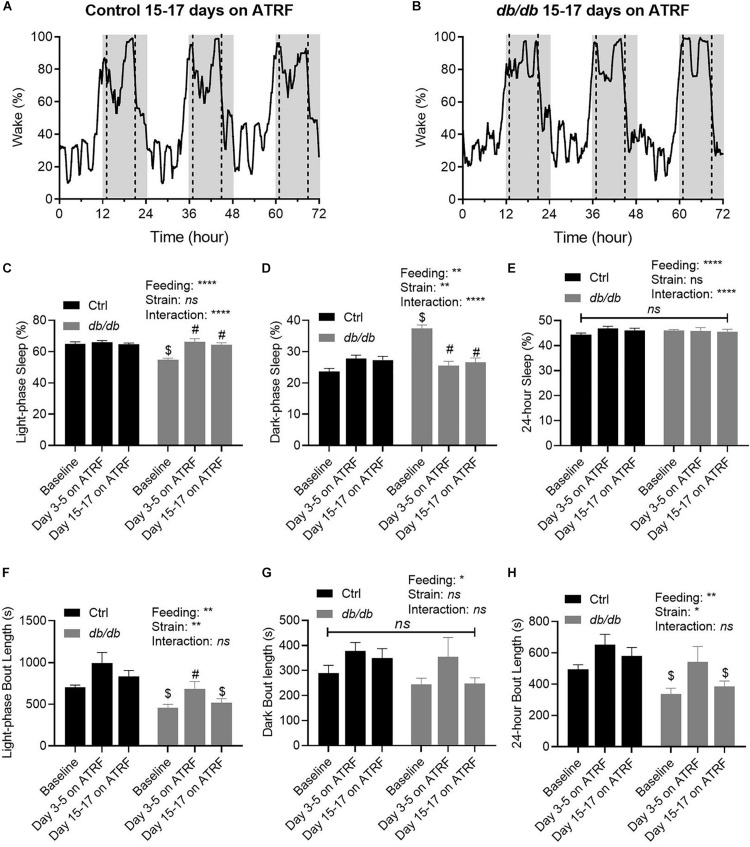
Time-dependent improvements of sleep-wake profile by ATRF in the control and *db/db* mice. Representative sleep-wake profiles in the control **(A)** and *db/db* mice **(B)** with 15–17 days of ATRF. The gray box indicates the dark-phase. The dotted vertical line indicates the time when food was added or withdrawn. **(C–E)** Average sleep time percent at baseline (ALF), 3–5 days and 15–17 days of ATRF during the light-phase **(C)**, dark-phase **(D)**, and over 24-h **(E)**. **(F–H)** Average bout length at baseline (ALF), 3–5 days, and 15–17 days of ATRF during the light-phase **(F)**, dark-phase **(G),** and over 24-h **(H)**. Control, *n* = 6–8; *db/db*, *n* = 6–7. ^$^*p* < 0.05 when compares with control. ^#^*p* < 0.05 when compares with baseline. ^∗^*p* < 0.05; ^∗∗^*p* < 0.01; ^*⁣*⁣**^p < 0.0001; *ns*, no significant.

To investigate further whether the improvement of the sleep-wake oscillation in the *db/db* mice would be maintained after the initial 5 days, we monitored the sleep-wake cycle in the two groups of mice on ATRF for the 15th through 17th days. As shown in Figures 4A,B, the sleep-wake cycle remained very similar between the control and *db/db* mice after the 17 days on ATRF, indicating the improvement was maintained at least for 17 days, the longest time monitored. The light-phase sleep percent remained at similar low levels on day 3–5 and on day 15–17 (Figure 4C and Supplementary Tables S5, S7), the dark-phase sleep percent remained at similar high levels on day 3–5 and on day 15–17 (Figure 4D and Supplementary Tables S5, S7), and the 24-h sleep percent remain unchanged (Figure 4E and Supplementary Tables S5, S7). Interestingly, while the dark-phase sleep bout length remained unchanged (Figure 4G and Supplementary Tables S6, S8), the increased sleep bout length during the light-phase, and 24-h observed during the initial 5 days seemed to be lost by day 15 to 17 (Figures 4F,H and Supplementary Tables S6, S8).

To determine whether the ATRF-induced sleep-wake improvement is associated with metabolic improvements in the *db/db* mice, we measured the body weight in *db/db* and control mice at baseline and after 18 days of ATRF: *db/db* mice body weight was decreased from 41.7 ± 0.9 g to 34.9 ± 1.1 g (*p* < 0.0001); however, the body weight of the control mice remained unchanged (28.1 ± 0.5 g vs. 29.1 ± 0.8 g, *p* > 0.05). The non-fasting blood glucose level measured at ZT20 was not significantly altered by ATRF in the *db/db* or control mice (mg/dl): 625.8 ± 27.0 vs. 665.3 ± 34.1, *p* > 0.05 in *db/db* mice and 136.5 ± 6.2 vs. 118.5 ± 3.4, *p* > 0.05 in the control mice.

## Discussion

In the present study, we used a non-invasive piezoelectric system to monitor the daily sleep-wake rhythm in the type 2 diabetic *db/db* mice and control mice under ALF and then under the active time-restricted feeding (ATRF) paradigm. The findings are (1) Compared with the controls, the *db/db* mice exhibited altered daily sleep-wake rhythms: during the light-phase, the sleep percent and sleep bout length were reduced, while during the active dark-phase, the sleep percentage but not bout length were increased, and (2) ATRF restored the sleep-wake pattern in the *db/db* mice to become similar to that of the control strain.

Previous human and animal studies have provided a great deal of evidence to demonstrate the effects of meal timing on metabolic health. However, very few studies have examined the effects of ATRF on sleep. Interestingly, previous investigations of time-restricted feeding on sleep were mostly focused on whether dissociation between feeding and circadian time would affect sleep-wake states. Animal studies showed that when the feeding time is restricted to the normal inactive-phase, the diurnal distribution of REM and NREM sleep is attenuated (Mouret and Bobillier, 1971; Roky et al., 1999; Mieda et al., 2004; Szentirmai et al., 2009). In contrast, experimental investigations of ATRF on sleep-wake rhythms are rare. One previous study in *Drosophila* demonstrated that ATRF decreases daytime sleep and increases nighttime sleep (Gill et al., 2015). Another study showed that ATRF improves sleep-wake timing in a Huntington’s Disease mouse model (Wang et al., 2018). In the present study, we first reported that ATRF is able to improve the sleep-wake cycle in diabetic *db/db* mice by increasing daytime sleep and decreasing nighttime sleep. However, due to the limitation of the piezoelectric system, it is unknown whether ATRF improves the rhythm of NREM EEG delta power, a marker of sleep homeostasis. However, the improved diurnal sleep-wake ratio (more sleep in the light period, less sleep in the dark period) as is typical in most mice, along with the reduced ultradian sleep-wake cycle at night in *ad lib db/db* mice, suggest improved sleep homeostatic function.

Sleep disturbance is common in diabetes. People with diabetes are more likely to experience sleep disturbance than those without (Lamond et al., 2000; Skomro et al., 2001; Nakanishi-Minami et al., 2012; Plantinga et al., 2012; Medeiros et al., 2013; Lecube et al., 2016). Compared to the large number of human studies, investigations of sleep-wake states in animals have been limited, and probably because of the difficulties in monitoring sleep in small animals. The present study used a non-invasive piezoelectric system to monitor sleep-wake states in the *db/db* mice. Since *db/db* mice are overweight and move less than smaller mice, it is possible that the PiezoSleep system might mis-score *db/db* mice more than normal mice. However, the *db/db* mice still display postural adjustments and subtle head movements for olfactory sampling and other needs that provide the same disruption of the breath signal to score quiet wake vs. sleep with good accuracy. Importantly, the *db/db* sleep data in this paper matches well with previous publications using the “gold standard” of EEG/EMG in *db/db* or *ob/ob* mice (Laposky et al., 2008; Silvani et al., 2009) showing similar dysregulation of sleep-wake cycles. The previous studies showed that the total sleep time, the dark-phase NREM, and REM sleep were all increased, whereas the light-phase REM sleep was decreased in *db/db* and *ob/ob* mice compared to wildtype mice (Laposky et al., 2008; Silvani et al., 2009). Although the piezoelectric system used in the current study cannot distinguish between NREM and REM sleep, we observed increased night-phase sleep and decreased light-phase sleep in *db/db* mice compared to the heterozygous control *db/* + mice. However, the total sleep time was not significantly different between *db/db* and *db/*+ mice. The discrepancy may be due to the different control mice used. The *db/*+ mice, which carry one allele of leptin receptor mutation, have normal body weight and blood glucose, but higher fat mass and plasma leptin levels relative to wildtype mice (Chung et al., 1998), and develop gestational diabetes during pregnancy (Kaufmann et al., 1981; Ishizuka et al., 1999). Therefore, it is possible that the one allele of leptin receptor mutation eliminates the total sleep time difference between *db/db* and *db/*+ mice. For the sleep bout length, the previous study showed that the light-phase and 24-h sleep bout length of NREM sleep were shortened in *db/db* mice compared to wildtype mice whereas no difference was found in REM sleep, which indicates the summed sleep bout length was shortened during light-phase and 24-h sleep. Consistent with that, we also detected similar alternations of sleep bout length in *db/db* mice. We also observed the unusual fragmentation of sleep and wake during the dark period (“spike” pattern), which demonstrated consistency between our study and prior EEG/EMG research.

The mechanisms underlying diabetes-induced disruption of sleep-wake states in *db/db* mice remain elusive. One possibility is diabetes-associated metabolic problems, such as obesity and impaired glucose control, contribute to the irregularity of sleep-wake states. Beside metabolic factors, another possibility is that diabetes disrupts clock gene circadian rhythms and thus the sleep-wake cycle. Interestingly, we and others have demonstrated that the mRNA and protein expressions of clock and clock-controlled genes are altered in *db/db* mice (Kudo et al., 2004; Caton et al., 2011; Su et al., 2012; Nernpermpisooth et al., 2015; Hou et al., 2019). In addition, alterations of clock genes are linked not only to the expected circadian disruptions but also to fundamental aspects of sleep homeostasis, suggesting clock genes may provide a previously uncharacterized “bridge” between *process c* and *process s* (Curie et al., 2013; Striz and O’Hara, 2013), the two process models of sleep regulation. Hence, it is possible that diabetes-associated clock gene alternations may contribute to sleep disturbances in *db/db* mice.

The precise mechanisms underlying the beneficial effects of ATRF on the *db/db* mouse’s sleep-wake cycle remain to be elucidated. The decrease in body weight likely contributes, however, we noted that the body weight of the *db/db* mice remained significantly higher than that of control mice after 18 days of ATRF. A decrease in blood glucose does not seem to contribute, as the non-fasting blood glucose was not significantly altered by ATRF in the *db/db* mice. In addition, ATRF-associated changes in the body temperature oscillation (Szentirmai et al., 2009) may also contribute to the improvement of sleep-wake cycle as body temperature modifies the sleep behavior (Liao, 2002). However, future studies are required to test this possibility. Evidence showed that the timing of food intake is a strong zeitgeber of peripheral clocks (Mendoza, 2007), and a previous study has demonstrated ATRF restores clock gene oscillation in *db/db* mouse liver (Kudo et al., 2004). We also demonstrated that ATRF-induced restoration of clock gene oscillation not only occurs in the liver but also in various other tissues (unpublished data). Hence it is possible that the restored rhythms of clock gene expression contribute to the improved sleep-wake patterns. In addition, we found that ATRF completely restored the sleep-wake cycle within 3 days suggesting the possibility of direct effects on sleep- and wake-promoting neuronal pathways or fundamental aspects of individual neurons (e.g., resting membrane potential). Further examinations on the activity of sleep- and wake-promoting neurons with ATRF are needed.

One limitation of the current study is that only the *db/db* mouse model was used. Although the phenotype of disrupted sleep in the obese diabetic *db/db* mice resembles that of human patients, the etiology of diabetes is different. Interestingly, an increase of sleep during the active dark-phase has also been reported in the high-fat diet-induced obese mouse model (Jenkins et al., 2006). It will be valuable to determine whether ATRF would be effective in improving the sleep-wake pattern in this additional mouse model in the future. In addition, an added ALF session after the ATRF would have provided data to verify that the ATRF-induced sleep-wake cycle improvement is not a time-dependent effect and to determine whether the ATRF-induced improvement would sustain for some time or not after the feeding regimen returns to ALF.

## Conclusion

In conclusion, the current study demonstrated an attenuated rhythm of the sleep-wake cycle and shortened sleep bout length in *db/db* mice and that ATRF is able to restore a more normal sleep-wake cycle (and tended to lengthen the sleep bout length), indicating ATRF may serve as a therapeutic strategy for improving the disrupted sleep-wake patterns that are characteristic of diabetes.

## Data Availability

All datasets generated for this study are included in the manuscript and/or the Supplementary Files.

## Ethics Statement

The animal study was reviewed and approved by the University of Kentucky Animal Care and Use Committee.

## Author Contributions

TH performed the experiments. CW, SJ, and TH analyzed the data. TH, ZG, and MG contributed to the idea, experimental design, and writing and editing of the manuscript. BO’H contributed to the interpretation of the sleep–wake data and editing of the manuscript.

## Conflict of Interest Statement

BO’H is a co-founder and owner of Signal Solutions LLC that manufactures and sells the PiezoSleep system. However, BO’H was not involved in any of the data collection or production of the graphs and results of this manuscript. The remaining authors declare that the research was conducted in the absence of any commercial or financial relationships that could be construed as a potential conflict of interest.

## Supplementary Material

FIGURE S1 The sleep-wake profiles in the control and *db/db* mice with ALF and 1–5 days of ATRF. The sleep-wake profiles with 95% confidence interval in the control **(A)** and *db/db* **(B)** mice. The gray box indicates the dark-phase.

FIGURE S2 Lomb-Scargle Periodogram of the control and *db/db* mice with ALF and 3–5 days of ATRF. **(A–D)** Individual mouse periodogram of the control mice with ALF **(A)** or ATRF **(C)** and *db/db* mice with ALF **(B)** or ATRF **(D)**. **(E)** The amplitude of the peak period length. **(F)** The sum amplitude of period length less than 6 h. ^****^*p* < 0.0001; *ns*, no significant.

TABLE S1 The light-, dark-phase, and 24-h sleep percent in control and *db/db* mice with ALF (baseline), and 1–5 days of ATRF.

TABLE S2 The light-, dark-phase, and 24-h sleep bout length in control and *db/db* mice with ALF (baseline), and 1–5 days of ATRF.

TABLE S3 Non-parametric circadian rhythm analysis of ALF (baseline) and 3–5 days of ATRF in control and *db/db* mice.

TABLE S4 Within-subject comparisons of non-parametric circadian rhythm analysis between ALF (baseline) and 3–5 days of ATRF.

TABLE S5 The light-, dark-phase, and 24-h sleep percent in control and *db/db* mice with ALF (baseline), 3–5 days, and 15–17 days of ATRF.

TABLE S6 The light-, dark-phase, and 24-h sleep bout length in control and *db/db* mice with ALF (baseline), 3–5 days, and 15–17 days of ATRF.

TABLE S7 Within-subject comparisons of sleep percent between ALF (baseline), 3–5 days, and 15–17 days of ATRF.

TABLE S8 Within-subject comparisons of sleep bout length between ALF (baseline), 3–5 days, and 15–17 days of ATRF.
