# Supplementary figures and images for: Active Time-Restricted Feeding Improved Sleep-Wake Cycle in db/db Mice
Source: Front Neurosci. 2019 Sep 20;13:969. doi: 10.3389/fnins.2019.00969 (PMC6763589; doi:10.3389/fnins.2019.00969)

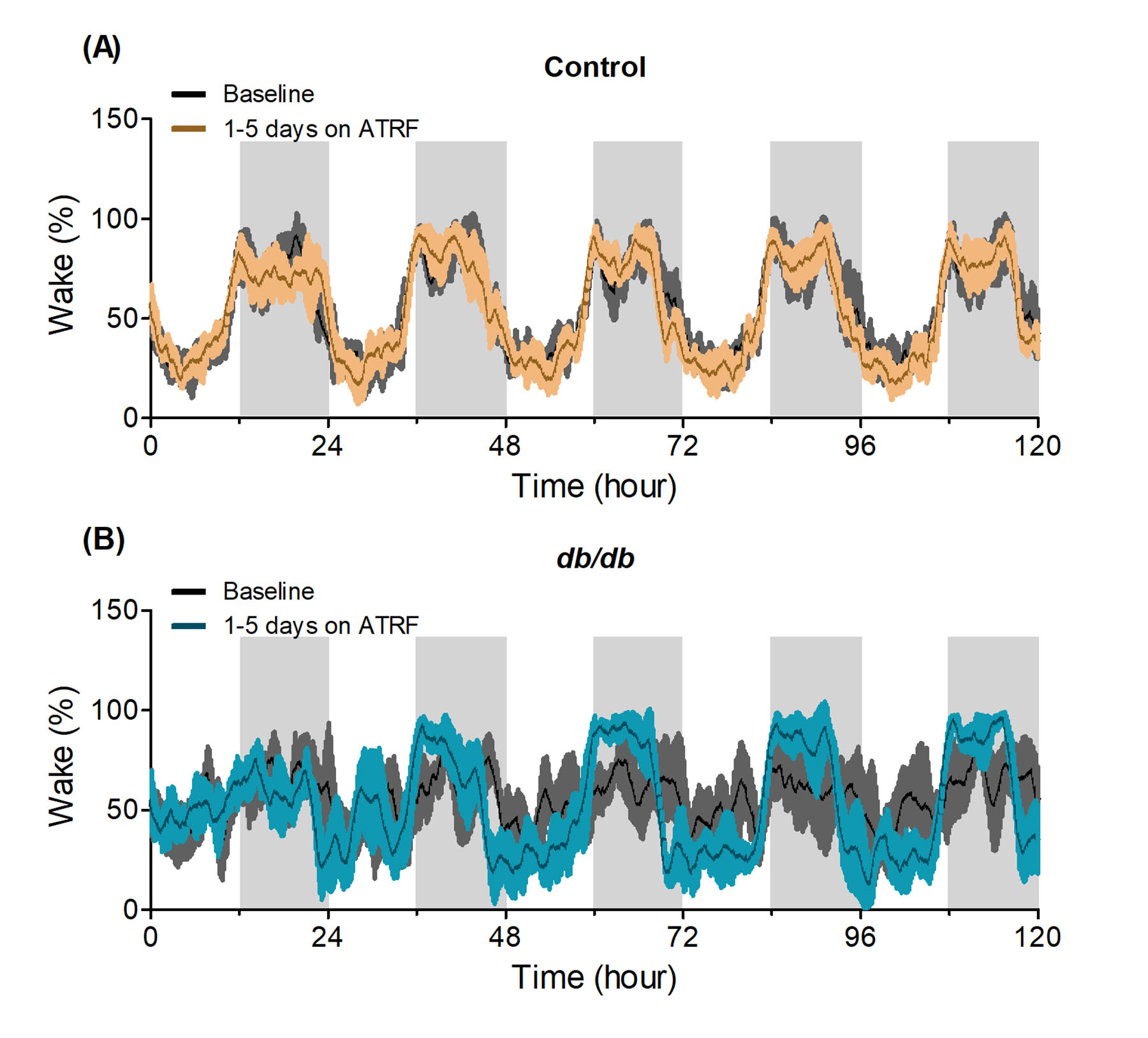

Supplement: FIGURE S1 — The sleep-wake profiles in the control and db/db mice with ALF and 1–5 days of ATRF. The sleep-wake profiles with 95% confidence interval in the control (A) and db/db (B) mice. The gray box indicates the dark-phase. [file Image_1.JPEG]

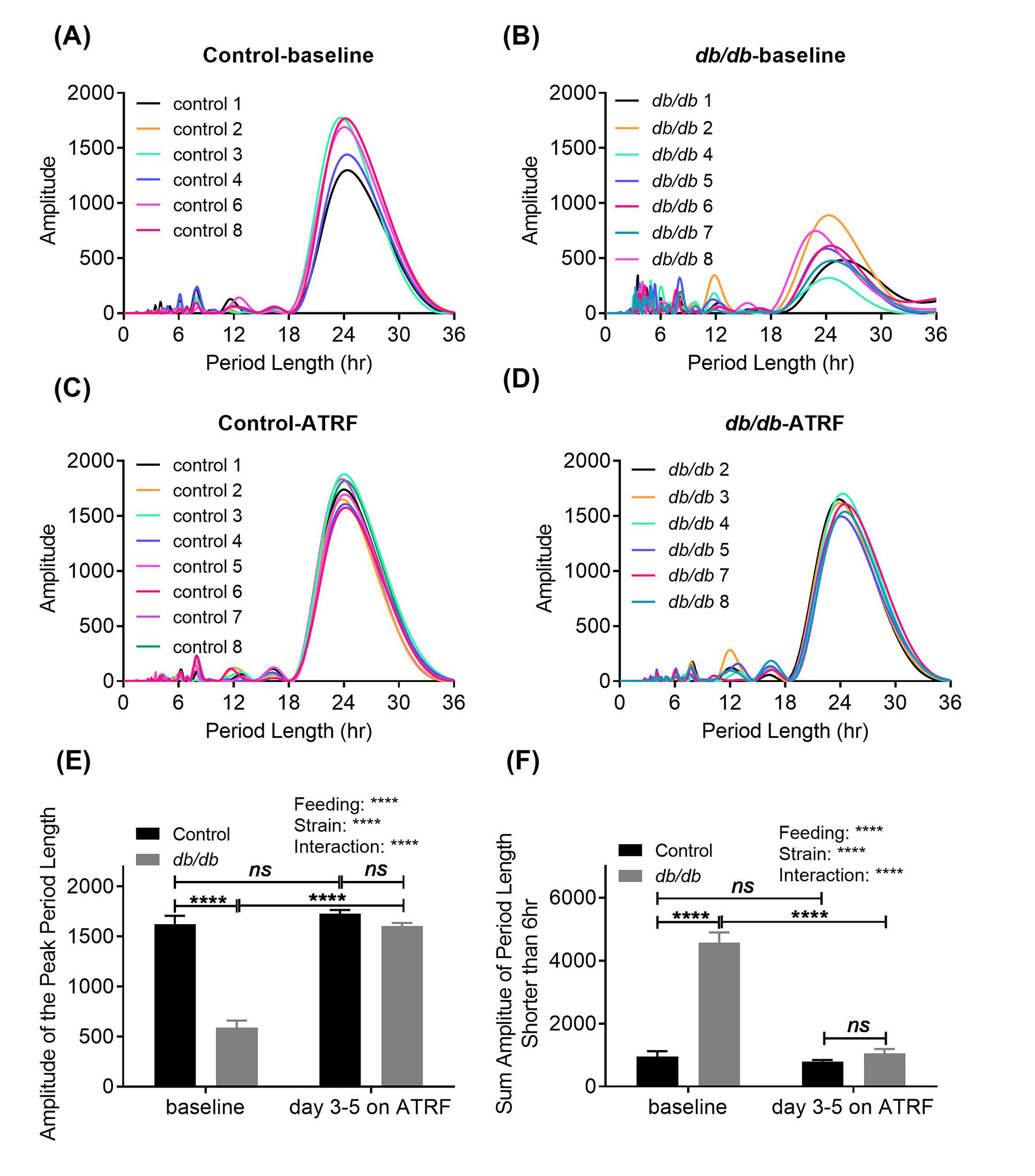

Supplement: FIGURE S2 — Lomb-Scargle Periodogram of the control and db/db mice with ALF and 3–5 days of ATRF. (A–D) Individual mouse periodogram of the control mice with ALF (A) or ATRF (C) and db/db mice with ALF (B) or ATRF (D). (E) The amplitude of the peak period length. (F) The sum amplitude of period length less than 6 h. ****p < 0.0001; ns, no significant. [file Image_2.JPEG]
