# Supplementary material for: Active Time-Restricted Feeding Improved Sleep-Wake Cycle in db/db Mice
Source: Front Neurosci. 2019 Sep 20;13:969. doi: 10.3389/fnins.2019.00969 (PMC6763589; doi:10.3389/fnins.2019.00969)
Supplement: TABLE S1 — The light-, dark-phase, and 24-h sleep percent in control and db/db mice with ALF (baseline), and 1–5 days of ATRF. [file Table_1.DOCX]

Table S1. The light-, dark-phase and 24-hour sleep percent in control and *db/db* mice with ALF (baseline) and 1-5 days of ATRF.

|  |  | **Control Mean±SD (%)** | ***Db/db***  **Mean±SD (%)** | **df** | ***t*** | ***p*** |
| --- | --- | --- | --- | --- | --- | --- |
| Light-phase Sleep (%) | Baseline | 64.9±3.33 | 54.8±2.56 | 9.34 | 6.06 | 0.0010 |
|  | Day 1 on ATRF | 61.4±3.75 | 51.9±3.65 | 11.09 | 4.80 | 0.0032 |
|  | Day 2 on ATRF | 63.9±5.23 | 55.6±4.78 | 11.42 | 3.07 | 0.0595 |
|  | Day 3 on ATRF | 65.0±3.20 | 64.7±8.13 | 6.17 | 0.08 | >0.9999 |
|  | Day 4 on ATRF | 65.8±4.13 | 67.1±3.70 | 11.52 | 0.59 | 0.9932 |
|  | Day 5 on ATRF | 67.1±4.79 | 67.0±6.65 | 8.70 | 0.01 | >0.9999 |
| Dark-phase Sleep (%) | Baseline | 23.7±2.39 | 37.5±2.77 | 10.99 | 9.63 | <0.0001 |
|  | Day 1 on ATRF | 27.8±5.66 | 44.1±4.46 | 11.94 | 6.03 | 0.0004 |
|  | Day 2 on ATRF | 23.8±3.03 | 34.4±5.51 | 7.27 | 4.23 | 0.0212 |
|  | Day 3 on ATRF | 28.5±3.09 | 27.2±3.49 | 10.11 | 0.75 | 0.9774 |
|  | Day 4 on ATRF | 27.0±3.45 | 25.4±6.58 | 7.06 | 0.57 | 0.9949 |
|  | Day 5 on ATRF | 27.8±3.60 | 24.0±5.56 | 8.07 | 1.47 | 0.6978 |
| 24-hour Sleep (%) | Baseline | 44.3±1.73 | 46.0±1.09 | 8.18 | 2.07 | 0.3578 |
|  | Day 1 on ATRF | 44.6±4.13 | 48.0±2.44 | 11.54 | 1.91 | 0.4009 |
|  | Day 2 on ATRF | 43.8±3.25 | 45.0±2.40 | 12.00 | 0.76 | 0.9762 |
|  | Day 3 on ATRF | 46.7±2.62 | 45.9±4.77 | 7.25 | 0.38 | 0.9994 |
|  | Day 4 on ATRF | 46.4±2.78 | 46.2±3.48 | 9.39 | 0.13 | >0.9999 |
|  | Day 5 on ATRF | 47.4±2.87 | 45.5±5.66 | 6.93 | 0.76 | 0.9780 |
