# Supplementary material for: Active Time-Restricted Feeding Improved Sleep-Wake Cycle in db/db Mice
Source: Front Neurosci. 2019 Sep 20;13:969. doi: 10.3389/fnins.2019.00969 (PMC6763589; doi:10.3389/fnins.2019.00969)
Supplement: TABLE S2 — The light-, dark-phase, and 24-h sleep bout length in control and db/db mice with ALF (baseline), and 1–5 days of ATRF. [file Table_2.DOCX]

Table S2. The light-, dark-phase and 24-hour sleep bout length in control and *db/db* mice with ALF (baseline) and 1-5 days of ATRF.

|  |  | **Control**  **Mean±SD (s)** | ***Db/db***  **Mean±SD (s)** | **df** | ***t*** | ***p*** |
| --- | --- | --- | --- | --- | --- | --- |
| Light-phase  Bout Length (s) | Baseline | 701.5±68.57 | 455.2±113.5 | 10.02 | 4.81 | 0.0043 |
|  | Day 1 on ATRF | 656.8±156.0 | 393.9±45.36 | 7.16 | 4.26 | 0.0213 |
|  | Day 2 on ATRF | 756.2±315.2 | 573.0±96.3 | 7.28 | 1.46 | 0.7090 |
|  | Day 3 on ATRF | 820.8±224.9 | 647.7±270.2 | 9.82 | 1.24 | 0.8112 |
|  | Day 4 on ATRF | 885.0±274.1 | 716.8±185.3 | 10.52 | 1.31 | 0.7707 |
|  | Day 5 on ATRF | 948.8±276.9 | 671.0±268.3 | 8.95 | 1.75 | 0.5199 |
| Dark-phase Bout Length (s) | Baseline | 289.3±77.29 | 244.2±66.0 | 9.96 | 1.12 | 0.8701 |
|  | Day 1 on ATRF | 316.7±133.1 | 393.0±121.5 | 10.94 | 1.08 | 0.8862 |
|  | Day 2 on ATRF | 274.5±70.47 | 405.8±216.6 | 5.91 | 1.42 | 0.7493 |
|  | Day 3 on ATRF | 350.8±119.3 | 409.5±245.9 | 6.98 | 0.53 | 0.9965 |
|  | Day 4 on ATRF | 340.2±57.44 | 351.1±214.8 | 5.61 | 0.12 | >0.9999 |
|  | Day 5 on ATRF | 363.3±45.61 | 282.3±133.7 | 6.00 | 1.42 | 0.7501 |
| 24-hour Bout Length (s) | Baseline | 495.7±69.43 | 337.0±97.26 | 10.71 | 3.42 | 0.0352 |
|  | Day 1 on ATRF | 478.7±144.3 | 387.1±58.01 | 8.13 | 1.54 | 0.6521 |
|  | Day 2 on ATRF | 499.1±173.9 | 462.2±70.80 | 8.18 | 0.51 | 0.9971 |
|  | Day 3 on ATRF | 574.1±170.6 | 536.7±269.5 | 8.22 | 0.29 | 0.9999 |
|  | Day 4 on ATRF | 583.8±107.3 | 529.8±220.0 | 7.00 | 0.55 | 0.9959 |
|  | Day 5 on ATRF | 630.6±102.0 | 454.7±191.7 | 5.63 | 1.87 | 0.5158 |
