# Supplementary material for: Active Time-Restricted Feeding Improved Sleep-Wake Cycle in db/db Mice
Source: Front Neurosci. 2019 Sep 20;13:969. doi: 10.3389/fnins.2019.00969 (PMC6763589; doi:10.3389/fnins.2019.00969)
Supplement: TABLE S3 — Non-parametric circadian rhythm analysis of ALF (baseline) and 3–5 days of ATRF in control and db/db mice. [file Table_3.DOCX]

Table S3. Non-parametric circadian rhythm analysis of ALF (baseline) and 3-5 days of ATRF in control and *db/db* mice.

|  |  | **Control**  **Mean±SD** | ***Db/db***  **Mean±SD** | **df** | ***t*** | ***p*** |
| --- | --- | --- | --- | --- | --- | --- |
| Interdaily Stability | Baseline | 0.9±0.02 | 0.6±0.07 | 23 | 14.77 | <0.0001 |
|  | Day 3-5 on ATRF | 0.9±0.02 | 0.9±0.05 | 23 | 1.64 | 0.2161 |
| Intradaily Variability | Baseline | 0.3±0.08 | 1.0±0.14 | 23 | 14.17 | <0.0001 |
|  | Day 3-5 on ATRF | 0.3±0.05 | 0.3±0.08 | 23 | 0.29 | 0.9507 |
| L5 Average | Baseline | 27.6±3.14 | 38.9±2.98 | 23 | 3.58 | 0.0032 |
|  | Day 3-5 on ATRF | 23.7±2.50 | 23.4±7.69 | 23 | 0.16 | 0.9838 |
| L5 Start | Baseline | 2.0±0.48 | 2.4±0.83 | 23 | 1.68 | 0.2021 |
|  | Day 3-5 on ATRF | 8.7±10.32 | 11.7±10.90 | 23 | 2.34 | 0.0526 |
| M10 Average | Baseline | 79.5±3.24 | 64.0±2.59 | 23 | 8.02 | <0.0001 |
|  | Day 3-5 on ATRF | 80.6±4.34 | 86.2±3.26 | 23 | 2.96 | 0.0141 |
| M10 Start | Baseline | 11.2±0.15 | 10.1±1.60 | 23 | 3.62 | 0.0029 |
|  | Day 3-5 on ATRF | 11.1±0.13 | 10.9±0.20 | 23 | 0.36 | 0.922 |
| Relative Amplitude | Baseline | 0.5±0.05 | 0.2±0.05 | 23 | 4.75 | 0.0002 |
|  | Day 3-5 on ATRF | 0.5±0.04 | 0.6±0.13 | 23 | 0.70 | 0.7403 |
| Diurnal Wake Ratio | Baseline | 5.1±0.93 | 3.1±0.53 | 22 | 3.35 | 0.0057 |
|  | Day 3-5 on ATRF | 6.5±0.93 | 6.0±1.77 | 22 | 0.69 | 0.7486 |
| Amplitude of the Peak Period Length | Baseline | 1622.8±203.38 | 558.2±188.33 | 23 | 12.20 | <0.0001 |
|  | Day 3-5 on ATRF | 1725.3±111.50 | 1602.9±75.18 | 23 | 1.49 | 0.2765 |
| Sum of Amplitude less than 6hr | Baseline | 945.5±441.59 | 4570.1±861.37 | 23 | 12.57 | <0.0001 |
|  | Day 3-5 on ATRF | 788.2±163.00 | 1057.8±335.74 | 23 | 0.91 | 0.6058 |
