# Supplementary material for: Active Time-Restricted Feeding Improved Sleep-Wake Cycle in db/db Mice
Source: Front Neurosci. 2019 Sep 20;13:969. doi: 10.3389/fnins.2019.00969 (PMC6763589; doi:10.3389/fnins.2019.00969)
Supplement: TABLE S4 — Within-subject comparisons of non-parametric circadian rhythm analysis between ALF (baseline) and 3–5 days of ATRF. [file Table_4.DOCX]

Table S4. Within-subject comparisons of non-parametric circadian rhythm analysis between ALF (baseline) and 3-5 days of ATRF.

|  | **Control** | | | | ***db/db*** | | | |
| --- | --- | --- | --- | --- | --- | --- | --- | --- |
| Baseline vs. day 3-5 on ATRF | df | *t* | Δ (%) | *p* | df | *t* | Δ (%) | *p* |
| Interdaily Stability | 9 | 0.70 | 1.7 | 0.7538 | 9 | 14.32 | 55.2 | <0.0001 |
| Intradaily Variability | 9 | 0.13 | 4.1 | 0.9902 | 9 | 14.01 | -70.2 | <0.0001 |
| L5 Average | 9 | 4.24 | -14.9 | 0.0044 | 9 | 12.84 | -33.6 | <0.0001 |
| L5 Start | 9 | 0.08 | 24.3 | 0.9957 | 9 | 0.70 | 2459.6 | 0.7505 |
| M10 Average | 9 | 0.61 | 1.7 | 0.8057 | 9 | 11.79 | 33.5 | <0.0001 |
| M10 Start | 9 | 0.19 | -0.0 | 0.9783 | 9 | 4.21 | -0.1 | 0.0046 |
| Relative Amplitude | 9 | 5.10 | 13.8 | 0.0013 | 9 | 21.23 | 131.8 | <0.0001 |
| Diurnal Wake Ratio | 9 | 2.74 | 32.8 | 0.0453 | 9 | 5.30 | 110.5 | 0.001 |
| Amplitude of the Peak Period Length | 9 | 1.26 | 6.9 | 0.4232 | 9 | 12.11 | 203.0 | <0.0001 |
| Sum of Amplitude less than 6hr | 9 | 0.62 | -0.1 | 0.7963 | 9 | 12.99 | -0.8 | <0.0001 |
