# Supplementary material for: Active Time-Restricted Feeding Improved Sleep-Wake Cycle in db/db Mice
Source: Front Neurosci. 2019 Sep 20;13:969. doi: 10.3389/fnins.2019.00969 (PMC6763589; doi:10.3389/fnins.2019.00969)
Supplement: TABLE S5 — The light-, dark-phase, and 24-h sleep percent in control and db/db mice with ALF (baseline), 3–5 days, and 15–17 days of ATRF. [file Table_5.DOCX]

Table S5. The light-, dark-phase and 24-hour sleep percent in control and *db/db* mice with ALF (baseline), 3-5 days and 15-17 days of ATRF.

|  |  | **Control**  **Mean±SD (%)** | ***Db/db***  **Mean±SD (%)** | **df** | ***t*** | ***p*** |
| --- | --- | --- | --- | --- | --- | --- |
| Light-phase Sleep (%) | Baseline | 64.9±3.33 | 54.8±2.56 | 9.34 | 6.06 | 0.0005 |
|  | Day 3-5 on ATRF | 65.9±3.33 | 66.2±4.97 | 8.26 | 0.13 | 0.9990 |
|  | Day 15-17 on ATRF | 64.6±2.52 | 64.5±3.39 | 12.92 | 0.05 | >0.9999 |
| Dark-phase sleep (%) | Baseline | 23.7±2.39 | 37.5±2.77 | 10.99 | 9.63 | <0.0001 |
|  | Day 3-5 on ATRF | 27.8±3.12 | 25.5±3.54 | 10.08 | 1.26 | 0.5567 |
|  | Day 15-17 on ATRF | 27.4±3.23 | 26.6±3.897 | 13.53 | 0.43 | 0.9665 |
| 24-hour Sleep (%) | Baseline | 44.3±1.73 | 46.0±1.09 | 8.180 | 2.07 | 0.1986 |
|  | Day 3-5 on ATRF | 46.9±2.39 | 45.9±3.15 | 9.053 | 0.64 | 0.8997 |
|  | Day 15-17 on ATRF | 46.0±2.64 | 45.6±2.72 | 13.99 | 0.31 | 0.9859 |
