# Supplementary material for: Active Time-Restricted Feeding Improved Sleep-Wake Cycle in db/db Mice
Source: Front Neurosci. 2019 Sep 20;13:969. doi: 10.3389/fnins.2019.00969 (PMC6763589; doi:10.3389/fnins.2019.00969)
Supplement: TABLE S6 — The light-, dark-phase, and 24-h sleep bout length in control and db/db mice with ALF (baseline), 3–5 days, and 15–17 days of ATRF. [file Table_6.DOCX]

Table S6. The light-, dark-phase and 24-hour sleep bout length in control and *db/db* mice with ALF (baseline), 3-5 days and 15-17 days of ATRF.

|  |  | **Control**  **Mean±SD (s)** | ***Db/db***  **Mean±SD (s)** | **df** | ***t*** | ***p*** |
| --- | --- | --- | --- | --- | --- | --- |
| Light-phase bout length (s) | Baseline | 701.5±68.57 | 455.2±113.5 | 10.02 | 4.81 | 0.0021 |
|  | Day 3-5 on ATRF | 993.3±357.6 | 685.5±213.0 | 11.57 | 2.01 | 0.1928 |
|  | Day 15-17 on ATRF | 834.9±194.8 | 517.2±140.0 | 12.71 | 3.75 | 0.0076 |
| Dark-phase bout length (s) | Baseline | 289.3±77.29 | 244.2±66.0 | 9.96 | 1.12 | 0.6396 |
|  | Day 3-5 on ATRF | 378.0±94.82 | 355.3±187.2 | 6.93 | 0.27 | 0.9912 |
|  | Day 15-17 on ATRF | 350.2±105.2 | 247.5±65.98 | 11.77 | 2.34 | 0.1092 |
| 24-hour bout length (s) | Baseline | 495.7±69.43 | 337.0±97.26 | 10.71 | 3.42 | 0.0178 |
|  | Day 3-5 on ATRF | 653.0±185.8 | 542.1±242.1 | 9.12 | 0.93 | 0.7550 |
|  | Day 15-17 on ATRF | 580.1±155.3 | 386.0±98.35 | 11.84 | 2.99 | 0.0341 |
