# Supplementary material for: Active Time-Restricted Feeding Improved Sleep-Wake Cycle in db/db Mice
Source: Front Neurosci. 2019 Sep 20;13:969. doi: 10.3389/fnins.2019.00969 (PMC6763589; doi:10.3389/fnins.2019.00969)
Supplement: TABLE S7 — Within-subject comparisons of sleep percent between ALF (baseline), 3–5 days, and 15–17 days of ATRF. [file Table_7.DOCX]

Table S7. Within-subject comparisons of sleep percent between ALF (baseline), 3-5 days and 15-17 days of ATRF.

|  |  | **Control** | | | | ***db/db*** | | | |
| --- | --- | --- | --- | --- | --- | --- | --- | --- | --- |
|  |  | df | *t* | Δ (%) | *p* | df | *t* | Δ (%) | *p* |
| Light-phase Sleep (%) | Baseline vs. day 3-5 on ATRF | 5 | 0.80 | 2.5 | 0.8434 | 4 | 7.93 | 19.1 | 0.0041 |
|  | Baseline vs. day 15-17 on ATRF | 5 | 0.19 | 0.0 | 0.9973 | 6 | 8.01 | 16.6 | 0.0006 |
|  | Day 3-5 vs. day 15-17 on ATRF | 7 | 1.63 | -1.9 | 0.3786 | 5 | 1.02 | -2.4 | 0.7318 |
| Dark-phase Sleep (%) | Baseline vs. day 3-5 on ATRF | 5 | 2.83 | 17.3 | 0.1059 | 4 | 5.87 | -32.7 | 0.0125 |
|  | Baseline vs. day 15-17 on ATRF | 5 | 2.08 | 21.9 | 0.2520 | 6 | 7.13 | -30.0 | 0.0012 |
|  | Day 3-5 vs. day 15-17 on ATRF | 7 | 0.28 | -0.5 | 0.9904 | 5 | 0.76 | 9.8 | 0.8595 |
| 24-hour Sleep (%) | Baseline vs. day 3-5 on ATRF | 5 | 1.96 | 6.4 | 0.2876 | 4 | 0.20 | -1.9 | 0.9965 |
|  | Baseline vs. day 15-17 on ATRF | 5 | 1.12 | 5.5 | 0.6782 | 6 | 0.38 | -2.1 | 0.9777 |
|  | Day 3-5 vs. day 15-17 on ATRF | 7 | 1.10 | -1.8 | 0.6672 | 5 | 0.26 | 0.8 | 0.9929 |
