# Supplementary material for: Active Time-Restricted Feeding Improved Sleep-Wake Cycle in db/db Mice
Source: Front Neurosci. 2019 Sep 20;13:969. doi: 10.3389/fnins.2019.00969 (PMC6763589; doi:10.3389/fnins.2019.00969)
Supplement: TABLE S8 — Within-subject comparisons of sleep bout length between ALF (baseline), 3–5 days, and 15–17 days of ATRF. [file Table_8.DOCX]

Table S8. Within-subject comparisons of sleep bout length between ALF (baseline), 3-5 days and 15-17 days of ATRF.

|  |  | **Control** | | | | ***db/db*** | | | |
| --- | --- | --- | --- | --- | --- | --- | --- | --- | --- |
|  |  | df | *t* | Δ (%) | *p* | df | *t* | Δ (%) | *p* |
| Light-phase Sleep Bout (s) | Baseline vs. day 3-5 on ATRF | 5 | 1.97 | 47.2 | 0.2861 | 4 | 4.51 | 39.2 | 0.0319 |
|  | Baseline vs. day 15-17 on ATRF | 5 | 1.54 | 16.7 | 0.4592 | 6 | 1.22 | 14.4 | 0.6074 |
|  | Day 3-5 vs. day 15-17 on ATRF | 7 | 1.74 | -10.9 | 0.3331 | 5 | 1.83 | -24.5 | 0.3351 |
| Dart-phase Sleep Bout (s) | Baseline vs. day 3-5 on ATRF | 5 | 2.17 | 37.9 | 0.2269 | 4 | 1.75 | 24.9 | 0.3955 |
|  | Baseline vs. day 15-17 on ATRF | 5 | 2.27 | 21.7 | 0.2020 | 6 | 0.21 | 2.9 | 0.9961 |
|  | Day 3-5 vs. day 15-17 on ATRF | 7 | 0.96 | -5.9 | 0.7476 | 5 | 1.58 | -14.8 | 0.4406 |
| 24-hour Sleep Bout (s) | Baseline vs. day 3-5 on ATRF | 5 | 1.95 | 37.2 | 0.2917 | 4 | 2.85 | 42.3 | 0.1324 |
|  | Baseline vs. day 15-17 on ATRF | 5 | 1.44 | 14.8 | 0.5067 | 6 | 1.69 | 16.6 | 0.3684 |
|  | Day 3-5 vs. day 15-17 on ATRF | 7 | 1.53 | -8.6 | 0.4296 | 6 | 1.64 | -21.8 | 0.4107 |
